# Progression of Age-Related Macular Degeneration Among Individuals Homozygous for Risk Alleles on Chromosome 1 (*CFH-CFHR5*) or Chromosome 10 (*ARMS2/HTRA1*) or Both

**DOI:** 10.1001/jamaophthalmol.2021.6072

**Published:** 2022-02-03

**Authors:** Steffen Schmitz-Valckenberg, Monika Fleckenstein, Moussa A. Zouache, Maximilian Pfau, Christian Pappas, Jill L. Hageman, Elvira Agrón, Claire Malley, Tiarnan D. L. Keenan, Emily Y. Chew, Gregory S. Hageman

**Affiliations:** 1 Sharon Eccles Steele Center for Translational Medicine, John A. Moran Eye Center, Department of Ophthalmology & Visual Sciences, University of Utah, Salt Lake City; 2 Utah Retinal Reading (UREAD) Center, John A. Moran Eye Center, Department of Ophthalmology & Visual Sciences, University of Utah, Salt Lake City; 3 GRADE Reading Center and Department of Ophthalmology, University of Bonn, Bonn, Germany; 4 Division of Epidemiology and Clinical Applications, National Eye Institute, National Institutes of Health, Bethesda, Maryland

## Abstract

**Importance:**

Age-related macular degeneration (AMD) is a common cause of irreversible vision loss among individuals older than 50 years. Although considerable advances have been made in our understanding of AMD genetics, the differential effects of major associated loci on disease manifestation and progression may not be well characterized.

**Objective:**

To elucidate the specific associations of the 2 most common genetic risk loci for AMD, the *CFH*-*CFHR5* locus on chromosome 1q32 (Chr1) and the *ARMS2*/*HTRA1* locus on chromosome 10q26 (Chr10)—independent of one another and in combination—with time to conversion to late-stage disease and to visual acuity loss.

**Design, Setting, and Participants:**

This case series study included 502 individuals who were homozygous for risk variants at both Chr1 and Chr10 (termed Chr1&10-risk) or at either Chr1 (Chr1-risk) or Chr10 (Chr10-risk) and who had enrolled in Genetic and Molecular Studies of Eye Diseases at the Sharon Eccles Steele Center for Translational Medicine between September 2009 and March 2020. Multimodal imaging data were reviewed for AMD staging, including grading of incomplete and complete retinal pigment epithelium and outer retinal atrophy.

**Main Outcomes and Measures:**

Hazard ratios and survival times for conversion to any late-stage AMD, atrophic or neovascular, and associated vision loss of 2 or more lines.

**Results:**

In total, 317 participants in the Chr1-risk group (median [IQR] age at first visit, 75.6 [69.5-81.7] years; 193 women [60.9%]), 93 participants in the Chr10-risk group (median [IQR] age at first visit, 77.5 [72.2-84.2] years; 62 women [66.7%]), and 92 participants in the Chr1&10-risk group (median [IQR] age at first visit, 71.7 [68.0-76.3] years; 62 women [67.4%]) were included in the analyses. After adjusting for age and AMD grade at first visit, compared with 257 participants in the Chr1-risk group, 56 participants in the Chr1&10-risk group (factor of 3.3 [95% CI, 1.6-6.8]; *P* < .001) and 58 participants in the Chr10-risk group (factor of 2.6 [95% CI, 1.3-5.2]; *P* = .007) were more likely to convert to a late-stage phenotype during follow-up. This difference was mostly associated with conversion to macular neovascularization, which occurred earlier in participants with Chr1&10-risk and Chr10-risk. Eyes in the Chr1&10-risk group (median [IQR] survival, 5.7 [2.1-11.1] years) were 2.1 (95% CI, 1.1-3.9; *P* = .03) times as likely and eyes in the Chr10-risk group (median [IQR] survival, 6.3 [2.7-11.3] years) were 1.8 (95% CI, 1.0-3.1; *P* = .05) times as likely to experience a visual acuity loss of 2 or more lines compared with eyes of the Chr1-risk group (median [IQR] survival, 9.4 [4.1-* (asterisk indicates event rate did not reach 75%)] years).

**Conclusions and Relevance:**

These findings suggest differential associations of the 2 major AMD-related risk loci with structural and functional disease progression and suggest distinct underlying biological mechanisms associated with these 2 loci. These genotype-phenotype associations may warrant consideration when designing and interpreting AMD research studies and clinical trials.

## Key Points

**Question:**

What are the independent and combined associations of the 2 most common genetic risk loci for age-related macular degeneration (AMD)—chromosomes 1 (*CFH-CFHR5*) and 10 (*ARMS2/HTRA1*)*—*with disease progression?

**Findings:**

This case series study of 502 individuals found that, after adjusting for age and baseline AMD severity, compared with individuals with 2 risk alleles exclusively at *CFH-CFHR5,* the time to conversion to late-stage disease was shortest among carriers of 2 risk alleles at each of the *ARMS2/HTRA1* and *CFH-CFHR5* loci, followed by individuals with 2 risk alleles exclusively at *ARMS2/HTRA1*.

**Meaning:**

These results suggest that the differential association of the 2 major genetic risk loci with disease progression may be substantial and may warrant consideration in AMD clinical research.

## Teaser

This case series study investigates whether the 2 gene loci most commonly associated with AMD are jointly or independently associated with disease progression as assessed structurally and via visual acuity loss in older adults.

## Introduction

Age-related macular degeneration (AMD) remains the leading cause of irreversible vision loss among individual older than 55 years of age in Western countries.^1^ Despite extensive research efforts and several large-scale clinical trials, no effective treatment is yet available to stop or slow the progressive degeneration and atrophy of photoreceptors, the retinal pigment epithelium (RPE), and the choriocapillaris that occur in all forms of late-stage AMD.^2,3,4^

The underlying pathologic mechanisms of the clinical manifestation and progression of AMD are incompletely understood.^5,6^ Age-related macular degeneration is considered a complex multifactorial disease that is associated with both genetic and environmental risk factors. Genome-wide association studies have identified 52 genetic variants in 34 loci independently associated with AMD.^7,8,9^ The 2 loci most commonly associated with AMD are the complement factor H (*CFH*)–complement factor H–related 5 (*CFHR5*) extended region on chromosome 1q32 (Chr1 locus) and the age-related maculopathy susceptibility 2/high-temperature requirement factor A1 (*ARMS2*/*HTRA1*), 2 tightly linked genes located on chromosome 10q26 (Chr10 locus).^10,11,12,13,14^ Other common AMD-associated loci (excluding rare variants) are not nearly as penetrant. Although they likely modulate disease to some degree, the other loci have only minor effects when assessed in the absence of risk at *CFH-CFHR5* and *ARMS2/HTRA1*.^9,15^ Evidence suggests that the pathologic molecular pathways associated with the Chr1 and Chr10 loci are distinct and that their respective contributions to clinical AMD manifestations, progression, and response to anti–vascular endothelial growth factor treatment differ markedly.^16,17,18,19,20,21,22,23^ It is essential to understand these differences more thoroughly to improve patient care and outcomes and to design more effective gene pathway-targeted therapeutics against AMD.

In parallel with elucidating the genetic susceptibility of AMD, major advances in high-resolution retinal imaging have occurred.^24,25,26^ Although optical coherence tomography (OCT) imaging has been used routinely in the clinical management of AMD for several years, most genetic association and genotype-phenotype correlation analyses are still based on color fundus photography or have not yet applied recently developed definitions and findings of early features of atrophy as detected by OCT and fundus autofluorescence.^27,28,29,30^ Indeed, a growing body of evidence suggests that multimodal retinal imaging allows for a refined detection of characteristic phenotypic hallmarks of AMD, including the ability to detect conversion to atrophic late-stage AMD earlier.^28,30,31^

In this study, we sought to elucidate the specific associations of the *CFH-CFHR5* and *ARMS/HTRA1* loci with the course of disease in patients with AMD. To do so, rather than including individuals based on phenotype, we selected individuals based on genetic risk status on Chr1 and Chr10. Of a large, longitudinal case series of individuals with AMD, 3 distinct subgroups met the inclusion criteria. Participants in the Chr1-risk and Chr10-risk groups carried 2 risk alleles exclusively at the *CFH-CFHR5* or the *ARMS2/HTRA1* locus but not both loci, whereas participants in the Chr1&10-risk group carried 2 risk alleles at each of the loci. By applying a refined OCT-based staging of AMD manifestation, we evaluated the conversion to late-stage AMD phenotypes and the rate of visual acuity loss in those 3 subgroups (Chr1-risk, Chr10-risk, and Chr1&10-risk).

## Methods

### Participants and Study Procedures

The Genetic and Molecular Studies of Eye Diseases at the Sharon Eccles Steele Center for Translational Medicine is an ongoing observational study that recruits individuals and family members with or without ocular disease from clinics at the John A. Moran Eye Center, University of Utah, its ancillary satellite locations, and the local community. The overall cohort is composed of approximately 4500 participants. For the present analyses, we reviewed data of participants who had been enrolled between September 2009 and March 2020. Study procedures included the assessment of demographic characteristics, medical and ocular histories, multimodal retinal imaging data, and genotyping results for AMD-associated variants. Snellen visual acuity was documented as part of the clinical routine. This study followed the reporting guideline for case series. The study adheres to the tenets of the Declaration of Helsinki^32^ and was approved by the University of Utah Institutional Review Board. Each participant provided informed written consent prior to enrollment in the study. No incentives or compensation was offered or provided to study participants.

### Genetic Stratification and Participant Selection

The cohort was stratified into genetic groups using *CFH-CFHR5* and *ARMS2/HTRA1* diplotype combinations.^33^ Genetic groups were defined based on the risk-conferring variants rs1061170 (*CFH Y402H*) on Chr1 and rs10490924 (*ARMS2 A69S*) on Chr10 with the goal of isolating genetic AMD risk either at 1 of the loci or at both.^10,11,12,13,34^ Participants included in this study carried 2 risk alleles on Chr1 and no risk alleles on Chr10 (Chr1-risk group), 2 risk alleles on Chr10 without any risk alleles on Chr 1 (Chr10-risk group), or 2 risk alleles on both Chr 1 and Chr10 (Chr1&10-risk group) (eTable 1 in the Supplement). Further details on genotyping and phenotyping regarding participant selection are provided in the eMethods in the Supplement.

### Image Grading

Eyes were graded as having early AMD if there were medium-sized, dome-shaped elevations detected on OCT B-scans corresponding to drusen with diameters larger than 63 μm but 125 μm or less and not showing hyperreflective foci. If drusen were larger than 125 μm in diameter or eyes exhibited hyperreflective foci corresponding to hyperpigmentary changes, a grade of intermediate AMD was determined. Within the intermediate AMD cohort, we specifically identified eyes with large pigment epithelial detachments (ie, dome-shaped elevations >1000 μm in basal diameter and >100 μm in maximum heights). We included the recently introduced AMD grade of incomplete RPE and outer retinal atrophy (iRORA) as an intermediate AMD grade.^28,30^ Classification of late-stage exudative neovascular AMD was based on clinical documentation (ie, history of intravitreal anti–vascular endothelial growth factor injection and corresponding signs on imaging data). The time of conversion was defined as the earliest documentation of exudation due to neovascular AMD in an eye with a previous diagnosis of early or intermediate AMD. The presence and conversion to late-stage neovascular AMD were confirmed by reviewing all available imaging data and based on the presence of disease activity signs, including intraretinal or subretinal fluid or hemorrhage. Ten eyes with development of late-stage neovascular AMD over time were excluded from the survival analysis because the time of initial conversion could not be determined from the documentation. Late-stage atrophic AMD was called at the earliest observation of complete RPE and outer retinal atrophy (cRORA), as recently described by the Classification of Atrophy Meeting group.^30^ For all eyes with manifestations of cRORA, the total atrophic lesion size was quantified using semiautomated software to assess fundus autofluorescence images (RegionFinder software, Heidelberg Engineering) with the support of near-infrared reflectance–OCT imaging. If no fundus autofluorescence imaging data were available, lesion size was quantified by manual outlining lesion boundaries on serial near-infrared reflectance images. To explore and account for potential effects of unequally distributed visit intervals, we defined the parameter *T*con as the time interval between the last visit before the conversion visit and the visit with first recorded conversion. As *T*con tends to zero, the recorded date of conversion tends to the “true” conversion date (eMethods in the Supplement).

### Statistical Analysis

All data were analyzed with the R software, version 4.0.1 (R Foundation for Statistical Computing), using the packages survival, survminer, and coxme.^35,36,37,38^ Analyses were carried out using Cox proportional hazards models. Log-likelihood statistics were used to assess the association between covariates and survival times. A 2-sided *P* < .05 was considered statistically significant. Whenever necessary, *P* values were adjusted for multiple testing using a Bonferroni correction.

## Results

### Demographic Data

There were 317 participants in the Chr1-risk group (median [IQR] age at first visit, 75.6 [69.5-81.7] years; 193 women [60.9%] and 124 men [39.1%]), 93 participants in the Chr10-risk group (median [IQR] age at first visit, 77.5 [72.2-84.2] years; 62 women [66.7%] and 31 men [33.3%]), and 92 participants in the Chr1&10-risk group (median [IQR] age at first visit, 71.7 [68.0-76.3] years; 62 women [67.4%] and 30 men [32.6%]) who were eligible and included in this study. Their demographic and phenotypic characteristics are given in Table 1 and described in detail in the eResults and eTable 2 in the Supplement.

**Table 1.** Characteristics of Participants in the Chr1-Risk and Chr10-Risk Groups at the First Visit

Characteristic	Chr1-risk	Chr10-risk	Chr1&10-risk	*P* value	χ^2^
No. of participants	317	93	92		
Sex					
Female	193	62	62	.36^a^	2.02
Male	124	31	30		
Age at first visit, median (IQR), y					
All participants	75.6 (69.5-81.7)	77.5 (72.2-84.2)	71.7 (68.0-76.3)	<.001^b^	21.8
At risk (1 or 2 eyes with early or intermediate AMD) at first visit	74.9 (68.8-80.5)	74.3 (71.4-81.7)	69.3 (66.1-73.1)	<.001^b^	21.6
Follow-up time, median (IQR), y					
All eyes	4.8 (1.5-8.5)	4.1 (1.3-6.9)	3.0 (1.2-8.4)	.07^b^	5.3
At risk (early or intermediate AMD) at first visit	4.6 (1.3-8.3)	4.4 (2.3-6.9)	4.0 (1.5-9.7)	.90^b^	0.1
AMD grade at first visit (participants), No. (%)					
Early or intermediate AMD/no AMD for fellow eye	18 (5.7)	3 (3.2)	3 (3.3)	.003^a^	26.9
Early or intermediate AMD/other for fellow eye^c^	14 (4.4)	1 (1.1)	3 (3.3)		
Bilateral early or intermediate AMD	162 (51.1)	34 (36.6)	31 (34.1)		
Early or intermediate AMD/late AMD for fellow eye	63 (19.9)	20 (21.5)	19 (20.9)		
Late AMD/other for fellow eye^c^	8 (2.5)	5 (5.4)	4 (4.4)		
Bilateral late AMD	52 (16.4)	30 (32.3)	31 (34.1)		
AMD grade at first visit (eyes), No. (%)					
At risk (early or intermediate AMD)	419 (68.4)	92 (50.5)	87 (48.9)	.002^a^	21.2
Drusen <125 μm and no pigmentary changes	137 (22.3)	28 (15.4)	17 (9.6)		
Drusen ≥125 and <1000 μm with or without pigmentary changes	225 (36.7)	46 (25.3)	64 (36.0)		
Large PED (≥1000-μm basal diameter)	38 (6.2)	7 (3.8)	4 (2.2)		
iRORA	19 (3.1)	11 (6.0)	2 (1.1)		
Late-stage AMD	175 (28.5)	85 (46.7)	86 (48.3)	.86^a^	0.3
Atrophic, total atrophic lesion size <2.54 mm^2^	46 (7.5)	13 (7.1)	18 (10.1)		
Atrophic, total atrophic lesion size ≥2.54 mm^2^	24 (3.9)	18 (9.9)	15 (8.4)		
Neovascular AMD					
Newly diagnosed	33 (5.4)	18 (9.9)	19 (10.7)		
Previously diagnosed	72 (11.7)	36 (19.8)	34 (19.1)		
Other, No. (%)					
Other condition	13 (2.1)	4 (2.2)	5 (2.8)	NA^e^	NA^e^
Ungradable	6 (1.0)	1 (0.6)	0		
No. of eyes excluded^d^	7	2	1	NA^e^	NA^e^

Abbreviations: AMD, age-related macular degeneration; Chr1-risk, homozygous for risk variants at chromosome 1 without risk at chromosome 10; Chr10-risk, homozygous for risk variants at chromosome 10 without risk at chromosome 1; Chr1&10, homozygous for risk variants at chromosomes 1 and 10; iRORA, incomplete retinal pigment epithelium and outer retinal atrophy; NA, not applicable; PED, pigment epithelium detachment; RPE, retinal pigment epithelium.

^a^ Pearson χ^2^ test.

^b^ Kruskal-Wallis rank sum test.

^c^ Eyes were excluded if any manifestation of additional retinal disease (eg, branch retinal vein occlusion, retinal detachment) was present or if eyes had undergone any retinal surgery or laser treatment; visits prior to the date such events manifested were included.

^d^ Eyes were excluded because the time of initial conversion could not be determined (more than 5 years between the last visit before the conversion visit and the visit with first recorded conversion).

^e^ Numbers too small to perform meaningful statistical analysis.

In total, 194 eyes (95.0%) with recorded conversion satisfied a *T*con of 5 or less years, and 112 eyes (54.9%) had a *T*con of 12 or less months. Within the subset of eyes with both a *T*con of 5 or less years the median [IQR] values were (1.02 [0.27-2.05] years for Chr1-risk, 0.63 [0.27-1.26] years for Chr10-risk, and 0.41 [0.26-0.78] years for Chr1&10-risk; *P* = .12) and a *T*con of 12 or less months (0.29 [0.17-0.52] years for Chr1-risk, 0.33 [0.21-0.49] years for Chr10-risk, and 0.34 [0.23-0.52] years for Chr1&10-risk; *P* = .60), *T*con did not differ significantly among the 3 groups. When applying either the *T*con of 12 or less months or the *T*con of 5 or less years criterion in the subsequent survival analyses, similar results were obtained. We therefore report herein the data for a *T*con of 12 or less months in detail, and the data for a *T*con of 5 or less years are provided in eFigures 1 and 2 in the Supplement. The initial total atrophic lesion size in all eyes converting to atrophic AMD was similar between the Chr1-risk (median [IQR] area, 0.24 [0.16-0.39] mm^2^), Chr10-risk (median [IQR] area, 0.22 [0.15-0.49] mm^2^), and Chr1&10-risk (median [IQR] area, 0.16 [0.14-0.29] mm^2^) groups (*P* = .30).

### Risk of Conversion to Late-Stage AMD

**Figure 1.**
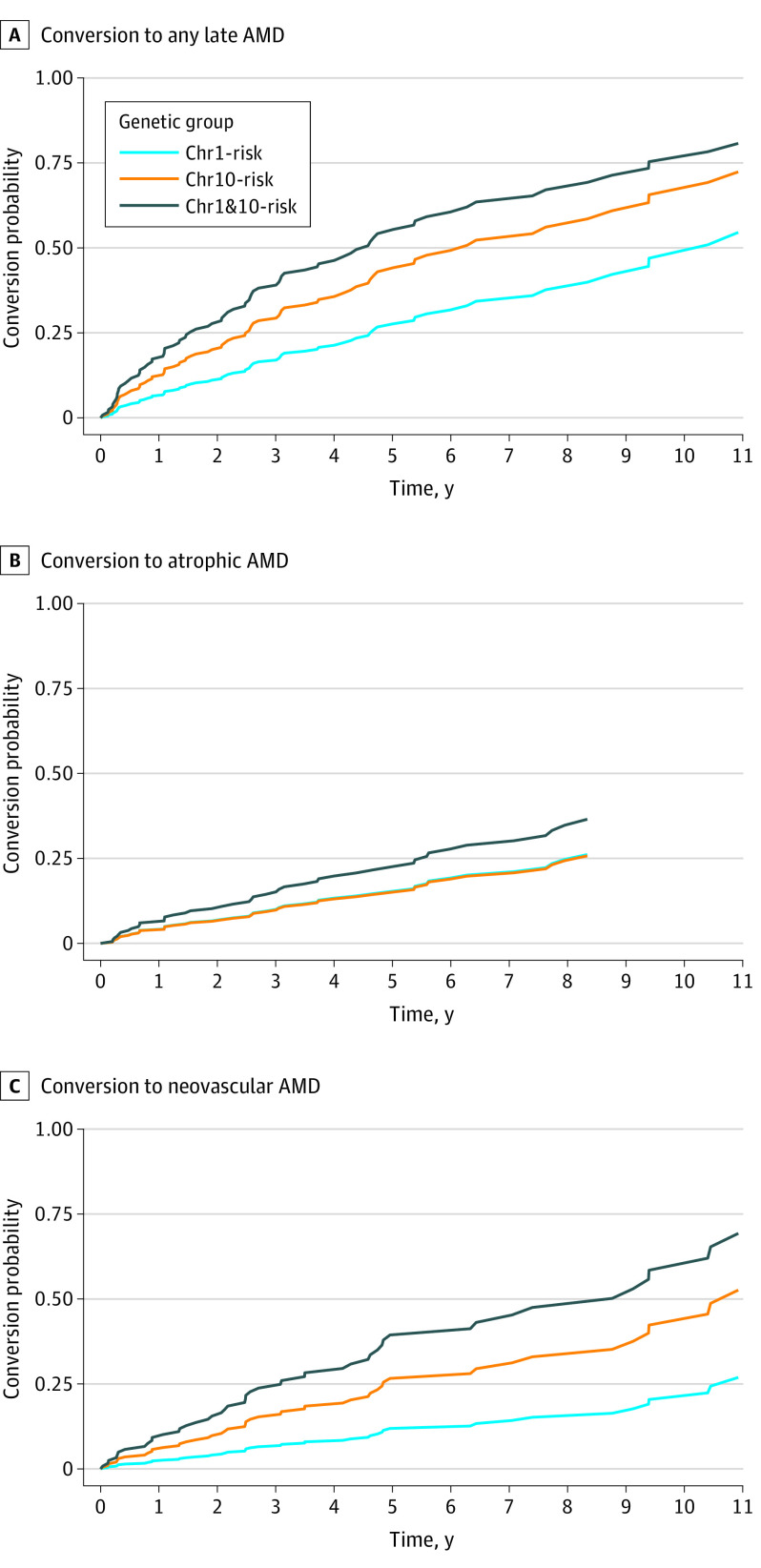
Survival Curves for Conversion to Any Late-Stage Age-Related Macular Degeneration (AMD) Phenotype Curves are shown for analyses including 1 eye per person and adjusted for age at first visit and refined AMD grade at first visit. Data are shown for participants with 12 or fewer months between the last visit before the conversion visit and the actual visit of conversion. Chr1-risk indicates homozygous for risk variants at chromosome 1 without risk at Chr10; Chr10-risk, homozygous for risk variants at chromosome 10 without risk at Chr1; and Chr1&10-risk, homozygous for risk variants at chromosomes 1 and 10.

Cox proportional hazards analyses indicated that eyes (selecting 1 eye per individual at risk) in the Chr1&10-risk group were 3.0 (95% CI, 1.7-5.4; *P* < .001) times as likely to convert to a late-stage phenotype than eyes in the Chr1-risk group, and eyes in the Chr10-risk group were 2.0 (95% CI, 1.1-3.6; *P* = .02) times as likely (median [IQR] survival time: 4.4 [1.4-9.4] years for Chr1&10-risk; 6.3 [2.5-11.0] years for Chr10-risk; and 10.4 [4.6-11.7] years for Chr1-risk) (Figure 1). The applied model was adjusted for both age and refined AMD grade at the time of the first visit because log-likelihood statistics revealed that these 2 factors were associated with risk to conversion, whereas sex was not. At the individual level (including both eyes of a person if both eyes showed early or intermediate AMD at the first visit), participants from the Chr1&10-risk group (factor of 3.3 [95% CI, 1.6-6.8]; *P* < .001) and Chr10-risk group (factor of 2.6 [95% CI, 1.3-5.1]; *P* = .007) were also more likely to convert to a late-stage phenotype compared with the Chr1-risk group (Table 2).

**Table 2.** HRs for Conversion to Late-Stage AMD, Shown at the Individual Level^a^

Variable	Any late-stage AMD		Atrophic AMD		Neovascular AMD	
	HR (95% CI)	*P* value	HR (95% CI)	*P* value	HR (95% CI)	*P* value
Genetic profile						
Chr1-risk	1 [Reference]		1 [Reference]		1 [Reference]	
Chr10-risk	2.6 (1.3-5.1)	.007	1.1 (0.5-2.5)	.76	2.8 (1.4-6.4)	.005
Chr1&10-risk	3.3 (1.6-6.8)	<.001	1.5 (0.7-3.2)	.35	4.4 (2.1-9.3)	<.001
Age at first visit	1.04 (1.0-1.1)	<.001	1.02 (1.0-1.1)	.21	1.03 (1.0-1.1)	.27
Refined AMD grade at first visit	-		-		-	
Medium-sized drusen	1 [Reference]		1 [Reference]		1 [Reference]	
Large drusen or pigmentary changes	7.5 (3.3-17.2)	<.001	12.5 (2.9-53.4)	<.001	2.7 (0.9-4.8)	.08
Large PED	16.8 (5.4-52.1)	<.001	18.4 (3.4-98.9)	<.001	3.3 (0.8-8.1)	.13
iRORA	49.1 (15.6-155)	<.001	73.7 (14.1-386)	<.001	4.0 (1.2-14.9)	.03

Abbreviations: AMD, age-related macular degeneration; Chr1-risk, homozygous for risk variants at chromosome 1 without risk at chromosome 10; Chr10-risk, homozygous for risk variants at chromosome 10 without risk at chromosome 1; Chr1&10, homozygous for risk variants at chromosomes 1 and 10; HR, hazard ratio; iRORA, incomplete retinal pigment epithelium and outer retinal atrophy; PED, pigment epithelium detachment.

^a^ Data include both eyes of 1 individual if both eyes showed early or intermediate AMD at first visit and data refer to persons with 12 or fewer months between the last visit before the conversion visit and the actual visit of conversion.

For both assessments (1 eye per individual vs both eyes included if both at risk), Cox proportional hazards modeling showed that the differences in time to conversion between genetic background groups appeared to be associated mostly with conversion to neovascular AMD rather than with conversion to atrophic AMD (hazard ratios [HRs], 2.8 [95% CI, 1.4-6.4] vs 1.1 [95% CI, 0.5-2.5] for Chr10-risk; 4.4 [95% CI, 2.1-9.3] vs 1.5 [95% CI, 0.7-3.2] for Chr1&10-risk at the individual level) (Figure 1 and Table 2). By contrast, the refined AMD grade at the first visit showed higher risk rates for conversion to late-stage atrophic AMD when controlling for the genetic group and age at first visit. For conversion into neovascular AMD, a significant association was only observed for iRORA (HR, 4.0 [95% CI, 1.2-14.9]; *P* = .03), while presence of large drusen and/or pigmentary changes (HR, 2.7 [95% CI, 0.9-4.8]; *P* = .08) and large pigment epithelium detachments (HR, 3.3 [95% CI, 0.8-8.1]; *P* = .13) were not statistically significant. For conversion to atrophic late-stage AMD, HRs reached higher levels, indicating an increasing risk with increasing drusen size category and presence of iRORA. Notably, the risk of developing atrophic AMD was increased by a factor of 12.5 (95% CI, 2.9-53.4; *P* < .001) in eyes with the refined AMD grade of large drusen or pigmentary changes compared with eyes with only medium-sized drusen at the first visit. For eyes with iRORA at the first visit, the risk was increased by a factor of 73.7 (95% CI, 14.1-386.0; *P* < .001). In addition, across the different AMD grades at risk for conversion, the survival time was shortest for eyes in the Chr1&10-risk group, followed by the Chr10-risk group, compared with Chr1-risk group, whereas consistently more advanced AMD phenotypes were also associated with shorter survival (Table 3). For example, the cumulative probability of conversion in eyes with medium-sized drusen in the Chr1-risk group did not reach 50% during follow-up, whereas the median (IQR) survival time was 0.9 (0.3-4.3) years for an eye with iRORA in the Chr1&10-risk group.

**Table 3.** Adjusted Median Survival Time For Conversion to Late-Stage Disease in Relation to the Refined AMD Stage at First Visit, Separately Shown For Both Genetic Groups^a^

Genetic group	Adjusted median (IQR)			
	Medium-sized drusen	Large drusen or pigmentary changes	Large PED	iRORA
Chr1-risk	NR (11.6 to *)	9.3 (7.4 to 11.6)	6.4 (4.3 to 11.8)	2.6 (1.6 to 9.4)
Chr10-risk	11.6 (10.9 to *)	5.0 (3.5 to 10.9)	3.7 (2.1 to *)	1.4 (0.8 to 4.0)
Chr1&10-risk	11.5 (9.4 to *)	3.5 (2.5 to 6.4)	2.6 (1.3 to *)	0.9 (0.3 to 4.3)

Abbreviations: asterisk (*), event rate did not reach 75%; AMD, age-related macular degeneration; Chr1-risk, homozygous for risk variants at chromosome 1 without risk at chromosome 10; Chr10-risk, homozygous for risk variants at chromosome 10 without risk at chromosome 1; Chr1&10, homozygous for risk variants at chromosomes 1 and 10; iRORA, incomplete retinal pigment epithelium and outer retinal atrophy; NR, event rate did not reach 50%; PED, pigment epithelium detachment.

^a^ Data are based on 1 eye per individual and are adjusted for age at first visit for persons with 12 or fewer months between the last visit before the conversion visit and the actual visit of conversion.

### Visual Acuity Loss

**Figure 2.**
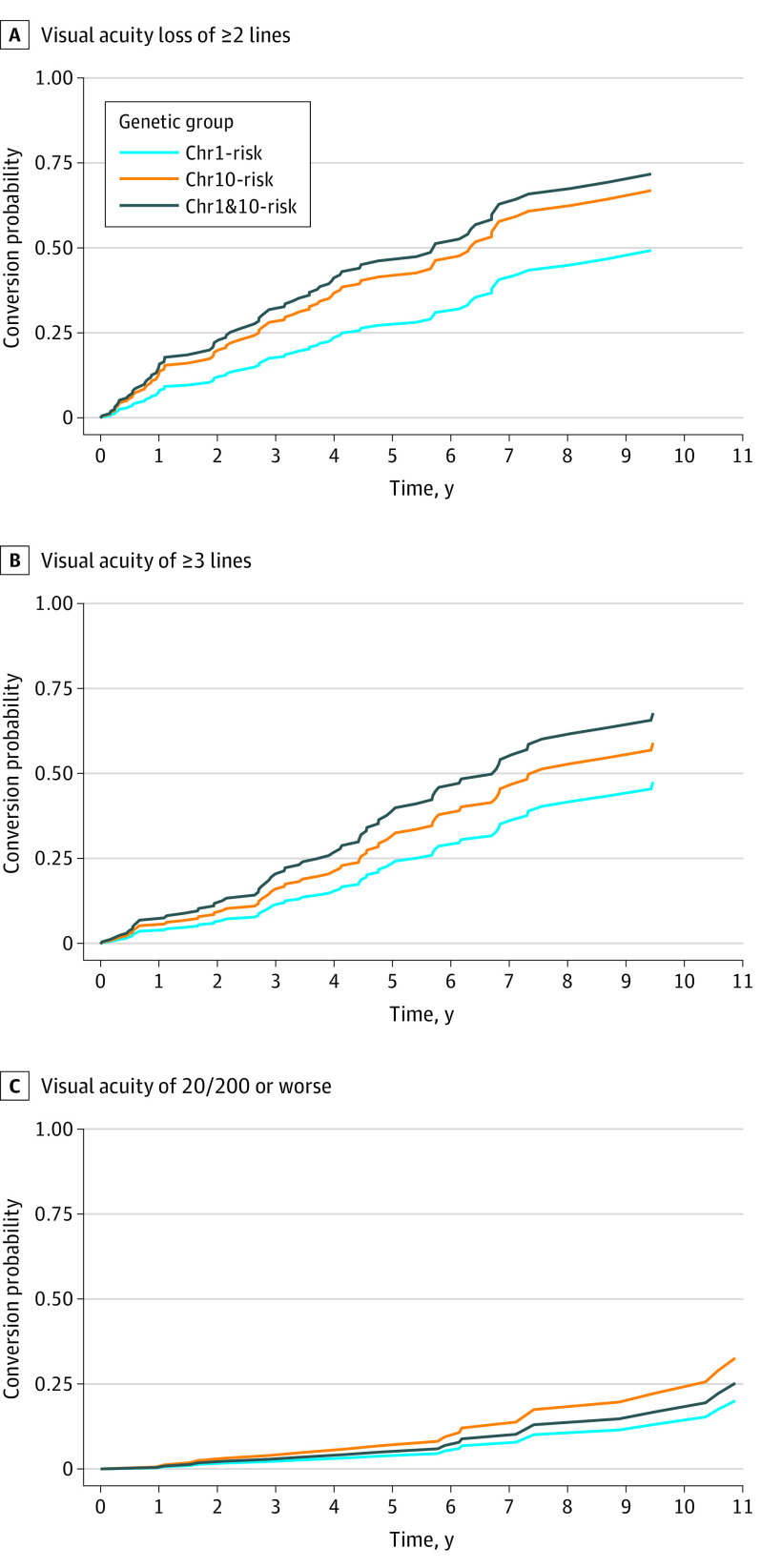
Survival Curves for Loss of Visual Acuity Curves are adjusted for age at first visit, refined age-related macular degeneration grade at first visit, and visual acuity at first visit. Data are shown for participants with 12 or fewer months between the last visit before the conversion visit and the actual visit of conversion. Chr1-risk indicates homozygous for risk variants at chromosome 1 without risk at Chr10; Chr10-risk, homozygous for risk variants at chromosome 10 without risk at Chr1; and Chr1&10-risk, homozygous for risk variants at chromosomes 1 and 10.

Based on log-likelihood analyses, age at first visit, visual acuity at first visit, and AMD grade at first visit were included in the Cox proportional hazards models as covariates. Sex and visual acuity in the fellow eye at the first visit did not show a significant association with visual loss. Survival analyses based on the eye with best visual acuity at the first visit showed that, compared with eyes from the Chr1-risk group, eyes from the Chr1&10-risk group were 2.1 (95% CI, 1.1-3.9; *P* = .03) times as likely to develop a visual acuity loss of 2 or more lines, and eyes from the Chr10-risk group were 1.8 (95% CI, 1.0-3.1; *P* = .05) times as likely (adjusted median [IQR] survival: 5.7 [2.2-11.1] years for Chr1&10-risk; 6.3 [2.7-11.3] years for Chr10-risk; and 9.4 [4.1-* (asterisk indicates the event rate did not reach 75%)] years for Chr1-risk) (Figure 2; eFigure 2 and eTable 3 in the Supplement). Median (IQR) survival times for visual acuity loss of 3 of more lines were 6.8 (3.7-11.3) years for Chr1&10-risk (*P* = .20) and 7.3 (4.4-*) years for Chr10-risk (*P* = .06), compared with 11.1 (5.4-*) years for Chr1-risk. For visual acuity loss of 20/200 or worse, the rate of events did not reach 50% in any group.

Evaluating visual acuity loss based on AMD grade at the first visit and controlling for the genetic subgroup, eyes with large drusen or pigmentary changes, as well as eyes with iRORA at the first visit, were at higher risk for visual acuity loss of both 2 or more lines and 3 or more lines compared with eyes with medium-sized drusen at the first visit (eTable 3 in the Supplement). Similar to the risk of conversion to late-stage AMD, the survival time for visual acuity loss of 2 or more lines was increasingly shorter for eyes in the Chr1&10-risk group, followed by the Chr10-risk group, across the different AMD grades at risk for conversion and compared with the Chr1-risk group (eTable 4 in the Supplement). For example, the median (IQR) survival time for visual acuity loss of 2 or more lines for an eye with medium-sized drusen in the Chr1-risk group was 13.44 (11.2-*) years compared with 2.6 (1.0-*) years for an eye with iRORA in the Chr1&10-risk group.

## Discussion

The findings of this case series study indicate that risk susceptibility solely at the AMD-associated Chr10 locus was associated with more rapid disease progression compared with genetic risk solely at Chr1. Carrying the cumulative susceptibility with homozygotes risk alleles at both of these loci was not only associated with an even more rapid disease progression but also with a younger age of conversion to late-stage disease (eResults in the Supplement). Both on an eye level and a person level, the Chr1&10-risk and Chr10-risk groups had a higher frequency of a late-stage phenotype at the first visit and a relatively higher number of conversions to late AMD. Cox proportional hazards ratio models that controlled for age and the refined AMD grade at the first visit indicated that eyes with early and intermediate AMD in Chr1&10-risk and Chr10-risk groups were 3.3 and 2.6 times more likely to develop late-stage AMD, respectively. These findings were consistent with visual acuity data, which showed the highest risk for functional decline in the Chr1&10-risk group, followed by the Chr10-risk group. Overall, it appears biologically plausible that individuals who have homozygous risk at both of the 2 major genetic AMD-associated loci manifest a more severe clinical disease pattern than individuals who have homozygous risk at 1 locus. Moreover, these results suggest that the underlying biological mechanisms of these 2 major AMD risk variants themselves are associated with distinct clinical disease manifestation, progression, and visual function.

Whereas Chr1-directed AMD is associated with high levels of complement activation at the RPE-Bruch membrane–choriocapillaris interface, the pathophysiology of Chr10-associated AMD is less well understood.^13,39^ That the difference between the 2 genetic groups in conversion and manifestation appears to be largely associated with neovascular AMD may indicate that the Chr10-risk is particularly associated with vascular abnormalities, including development of neovascular AMD. Previous reports have also suggested a stronger trend for the *ARMS2/HTRA1* locus than for the *CFH* locus for progression to neovascular AMD.^40,41^ Future studies using OCT angiography and postmortem histologic analyses may be helpful to further elucidate these findings.

In addition to genetic association, we confirmed that older age and a more severe AMD phenotype at the first visit were associated with increased risk for conversion.^42,43,44,45,46^ Compared with most previous analyses that used color fundus photography for AMD classification, we applied refined AMD grading based on OCT imaging and including early atrophic features, including iRORA and cRORA, allowing us to detect characteristic hallmarks of disease manifestation and progression with more detail and precision. The results of this approach are underscored by the large differences in risk of conversion and median survival times among refined AMD grades at the first visit, showing an increased relative risk as high as 73.7 for iRORA compared with medium-sized drusen. Furthermore, the median survival times ranged from not reaching a cumulative probability of 50% during follow-up to as short as 0.9 years. These findings support a key role for multimodal imaging in future genotype-phenotype assessments of AMD, particularly for the identification of high-risk eyes for disease progression.

### Limitations

The limitations of these analyses are that phenotypic data were not collected as part of a prospective, multicenter study with fixed-visit intervals but were based on a single-center, case series from a clinical practice setting with unequally distributed visit intervals. Attempts to address these limitations were included in our statistical approach, which considered several covariables. Major confounding in data collection were excluded. This does not apply to a potential sampling bias because the Genetic and Molecular Studies of Eye Diseases at the Sharon Eccles Steele Center for Translational Medicine should not be considered population based but rather a clinic-based population consisting of individuals seeking eye care (for various reasons) and their respective family members. In strict terms, these potential disease-ascertainment and family-history biases do not allow for the derivation of absolute risks for AMD progression in the general population. The analyses of visual acuity loss were not based on standard Early Treatment in Diabetic Retinopathy Study visual acuity assessments but relied on Snellen visual acuity that was documented as part of routine clinical assessment, the latter being considered less robust than the former.^47^ Furthermore, if eyes converted to late-stage neovascular AMD, it can be assumed that the course of visual acuity would depend on various factors, largely influenced by routine patient care and treatment regimen that are difficult to control in the current data set. Nevertheless, we showed that both Chr1&10-risk and Chr10-risk were—in addition to pure conversion—associated with higher risk of visual acuity loss. In addition, this study focused on 3 major and distinct genetic subgroups in the AMD spectrum. Although we appreciate that the genetics of AMD susceptibility are complex, we believe that this approach, which defined exclusive genetic risks at just 1 of the 2 major genetic variants (and having no risk at the other locus), enabled us to decipher distinct genetic correlations with phenotypic AMD characteristics. We propose that this method will guide future therapeutic approaches, especially those that are directed toward Chr1- or Chr10-mediated pathways and targets.

## Conclusions

The consistent genotype-phenotypic associations of the 2 major AMD-associated genetic loci with disease progression and visual function decline suggest that distinct loci-specific biological mechanisms exist and that these mechanisms can be characterized clinically. Validation in other cohorts and analyses of the association of these independent genetic diplotypes with other AMD manifestations appears warranted.

## Notes

Supplement. **eMethods.** Genotyping, Phenotyping and Statistical Analysis **eResults.** Demographic and Phenotypic Characteristics **eFigure 1.** Survival Curves for Conversion Data Based Upon *T_con_* ≤ 5 Years **eFigure 2.** Survival Curves for Loss of Visual Acuity Based Upon *T_con_* ≤ 5 Years **eTable 1.** Genetic Diplotypes Employed to Define the Chr1-Risk, Chr10-Risk and Chr1&10-Risk Groups **eTable 2.** Comparison of Longitudinal Data for Eyes at Risk at the First Visit Between Genetic Groups on an Eye Level **eTable 3.** Hazard Ratios for Visual Acuity Loss ≥ 2 Lines and ≥ 3 Lines **eTable 4.** Adjusted Median Survival Time For Visual Acuity Loss ≥ 2 Lines in Relation to the Refined AMD Stage at the First Visit eReferences
