# Supplementary material for: Progression of Age-Related Macular Degeneration Among Individuals Homozygous for Risk Alleles on Chromosome 1 (CFH-CFHR5) or Chromosome 10 (ARMS2/HTRA1) or Both
Source: JAMA Ophthalmol. 2022 Feb 3;140(3):252–60. doi: 10.1001/jamaophthalmol.2021.6072 (PMC8814975; doi:10.1001/jamaophthalmol.2021.6072)
Supplement: Supplement. — eMethods. Genotyping, Phenotyping and Statistical Analysis eResults. Demographic and Phenotypic Characteristics eFigure 1. Survival Curves for Conversion Data Based Upon Tcon ≤ 5 Years eFigure 2. Survival Curves for Loss of Visual Acuity Based Upon Tcon ≤ 5 Years eTable 1. Genetic Diplotypes Employed to Define the Chr1-Risk, Chr10-Risk and Chr1&10-Risk Groups eTable 2. Comparison of Longitudinal Data for Eyes at Risk at the First Visit Between Genetic Groups on an Eye Level eTable 3. Hazard Ratios for Visual Acuity Loss ≥ 2 Lines and ≥ 3 Lines eTable 4. Adjusted Median Survival Time For Visual Acuity Loss ≥ 2 Lines in Relation to the Refined AMD Stage at the First Visit eReferences [file jamaophthalmol-e216072-s001.pdf]

Supplementary Online Content

Schmitz-Valckenberg S, Fleckenstein M, Zouache MA, et al. Progression of age-related macular degeneration among individuals homozygous for risk alleles on chromosome 1 (CFH-CFHR5) or chromosome 10 (ARMS2/HTRA1) or both. *JAMA Ophthalmol*. Published online February 3, 2022. doi:10.1001/jamaophthalmol.2021.6072

eMethods. Genotyping, Phenotyping and Statistical Analysis

eResults. Demographic and Phenotypic Characteristics

eFigure 1. Survival Curves for Conversion Data Based Upon $T_{con} \leq 5$ Years

eFigure 2. Survival Curves for Loss of Visual Acuity Based Upon $T_{con} \leq 5$ Years

eTable 1. Genetic Diplotypes Employed to Define the Chr1-Risk, Chr10-Risk and Chr1&10-Risk Groups

eTable 2. Comparison of Longitudinal Data for Eyes at Risk at the First Visit Between Genetic Groups on an Eye Level

eTable 3. Hazard Ratios for Visual Acuity Loss ≥ 2 Lines and ≥ 3 Lines

eTable 4. Adjusted Median Survival Time For Visual Acuity Loss ≥ 2 Lines in Relation to the Refined AMD Stage at the First Visit

eReferences.

This supplementary material has been provided by the authors to give readers additional information about their work.

eMethods. Genotyping, Phenotyping, and Statistical Analysis

Genotyping

Genomic DNA was isolated from peripheral blood leukocytes, cheek swabs or saliva (QIAamp DNA Blood Maxi, Qiagen, Valencia, CA; Isohelix BFP-50 Plus DNA Isolation Kit, Cell Projects Ltd. Harrietsham, Kent, UK and Oragene OG-500 kit, DNA Genotek, Ottawa, ON, Canada). Genotyping was performed by TaqMan assays (Applied Biosystems, Foster City, California) using 10ng of template DNA in a 5µL reaction. A pre-designed assay, C_29934973_20, was used to genotype rs10490924. Genotyping of rs1061170 was performed using a custom assay designed using the manufacturer's design software. The thermal cycling conditions in the 384-well thermocycler (PTC-225, MJ Research) consisted of an initial hold at 95°C for 10 minutes, followed by 40 cycles of a 15-second 95°C denaturation step and a 1-minute 60°C annealing and extension step. Plates were read in the 7900HT Fast Real-Time PCR System (Applied Biosystems).

Phenotyping

All available imaging data of subjects included in this study were reviewed and graded at the Utah Retinal Imaging Reading Center (UREAD) by two retina specialists (SSV and MF). These included historical imaging data collected prior to enrollment in the prospective study, going as far back as to April 2001. A multimodal imaging approach, including color fundus photography, fluorescein angiography, indocyanine green angiography, FAF and OCT, and using spectral-domain OCT volume scans combined with confocal scanning laser ophthalmoscopy near-infrared (NIR) imaging (Spectralis HRA+OCT, Heidelberg Engineering GmbH, Germany as the base modality (if available) was employed for AMD grading. In total, 16,124 of combined OCT+NIR data sets were reviewed and graded (9,832 for *Chr1-risk* group, 3,305 for *Chr10-risk* group, 2,987 for

© 2022 Schmitz-Valckenberg S et al. *JAMA Ophthalmol.*

Chr1&10-risk group). Subjects had to be ≥ 50 years of age at the first diagnosis of AMD to be included in the analysis. Eyes were excluded if any manifestation of additional retinal disease (*e.g.* branch retinal vein occlusion, retinal detachment) was present or if eyes had undergone any retinal surgery and/or laser treatment. Visits prior to the data such events manifested were included.

Statistical Analyses

For survival analyses of risk of progression to late-stage AMD, we defined for each eye the first visit as the earliest visit available with imaging-based documentation of a clinical phenotype of AMD (as defined above). The timepoint of conversion was defined as the first occurrence of either late-stage atrophic or late-stage exudative AMD (as defined above). Conversion to atrophic and neovascular AMD were also evaluated separately. For these sub-group analyses, only eyes with conversion to the relevant phenotype (either atrophic or neovascular AMD were defined as events, while conversion to the other phenotype was censored. For all analyses, eyes were censored if no conversion had occurred at the last available visit. Further, eyes were censored at the timepoint of any potential confounding event. These included occurrence of other retinal diseases (*e.g.* branch retinal vein occlusion) or retinal surgery (*e.g.* retinal detachment surgery).

Survival analyses were performed using R and packages survival, survminer and coxme).[1-4] Analyses were carried out at both the eye and patient level (using both eyes of one subject as random effects) using Cox proportional hazard models. Log-likelihood statistics were used to determine if co-variates including gender and – as assessed at the first visit – age and AMD grade should be included. For the survival analyses at the eye level, the hazard ratio for only one eye per patient was used. If both eyes of a subject were at risk (both eyes had either early or intermediate AMD at the first visit), the following approach was defined for selecting the eye to be used: (1) the eye that

© 2022 Schmitz-Valckenberg S et al. *JAMA Ophthalmol.*

converted; (2) if both eyes converted, the one that converted later and; (3) if both eyes converted at the same timepoint, the one with the more severe AMD grade at the first visit. The first occurrence of a loss of VA ≥ 2 lines, ≥ 3 lines and to 20/200 or worse, as compared to the first visit in eyes at risk (*i.e.* either early or intermediate AMD at the first visit) was compared for the *Chr1&10-risk* and *Chr10-risk* groups to the *Chr1-risk*, respectively. We evaluated VA changes in the better seeing eye (as assessed as the first visit) if both eyes were at risk for conversion. Snellen VA was converted into logMAR VA. Similar to the analyses of the risk of conversion into late-stage AMD, Cox proportional hazard models with log-likelihood statistics for assessment of potential covariates were applied. The significance level for all statistical tests was set at $\alpha = 0.05$. All *p*-values generated are two-sided. When necessary, *p*-values were adjusted for multiple testing using a Bonferroni correction.

We assessed possible uncertainties in the exact conversion date (caused by unequally distributed times between visits) by introducing the parameter T_{con}, defined as the time interval between the last recorded visit prior to conversion to late-stage AMD and the date of the first recorded conversion, which was calculated for each eye with a recorded conversion. As T_{con} tends to zero, the recorded date of conversion tends to the “true” conversion date. The distribution of this parameter did not differ significantly between genetic groups when considering $T_{con} \leq 5$ years or $T_{con} \leq 12$ months ($p > 0.15$). We also assessed possible uncertainties in the exact conversion date by examining the total atrophic lesion size on the conversion visit. Differences between genetic groups were determined using linear mixed-effect models to account for measurements made in both eyes.

© 2022 Schmitz-Valckenberg S et al. *JAMA Ophthalmol.*

eResults. Demographic and Phenotypic Characteristics

Subjects at risk (*i.e.* one or two eyes with early/intermediate AMD) were younger in the *Chr1&10-risk* group (n = 56, median 69.3 years) at the first recorded visit as compared to those in the *Chr1-risk* (n = 257, 74.9 years) and *Chr10-risk* (n – 58, 74.3 years, $p <$ 0.001) groups (Table 1). Duration of follow-up for subjects at risk were similar between the three groups (4.0 years, 4.6 years and 4.4 years, $p = 0.9$). AMD staging at the first visit revealed significant differences between the three groups, showing overall earlier forms of disease in the *Chr1-risk* group as compared to the other two risk groups ($p =$ 0.003) (Table 1). Specifically, the proportion of subjects with bilateral early or intermediate AMD was higher in the *Chr1-risk* group (51.1% vs. 36.6% in *Chr10-risk* and 34.1% in *Chr1&10-risk* groups), while bilateral late-stage AMD was more prevalent in the *Chr1&10-risk* and *Chr10-risk* groups (34.1% and 32.3% vs. 16.4% in *Chr1-risk* group). At the eye level, the higher proportions of eyes with late-stage AMD in both the *Chr1&10-risk* and *Chr10-risk* groups were driven largely by a higher frequency of the neovascular form of the disease (29.8% and 29.7% vs. 17.1%, $p < 0.001$).

Over time, a comparatively larger number of conversions was observed in both the *Chr1&10-risk* and *Chr10-risk* groups; this was driven mainly by conversions to neovascular disease, as compared to atrophic AMD (eTable 2). The age at time of conversion was similar between the *Chr1-risk* (80.6 years) and *Chr10-risk* (82.6 years) genetic groups. However, it was markedly younger in the *Chr1&10-risk* group (73.6 years) ($p < 0.001$). Out of the 394 eyes at risk of conversion at the first visit, 34 (8.6%) were lost to follow-up because of death. Of these 34 eyes, there were 26 (9.7%) in the *Chr1-risk* group, 5 (9.8%) in the *Chr10-risk* group and 3 (6.3%) in the *Chr1&10-risk* group, showing a younger age of death in the latter (73.3 years), as compared to the other two subgroups (82.2 years in *Chr1-risk* and 85.4 years in *Chr10-risk*, $p = 0.02$).

© 2022 Schmitz-Valckenberg S et al. *JAMA Ophthalmol.*

eFigure 1. Survival Curves for Conversion Data Based Upon T_{con} ≤5 Years

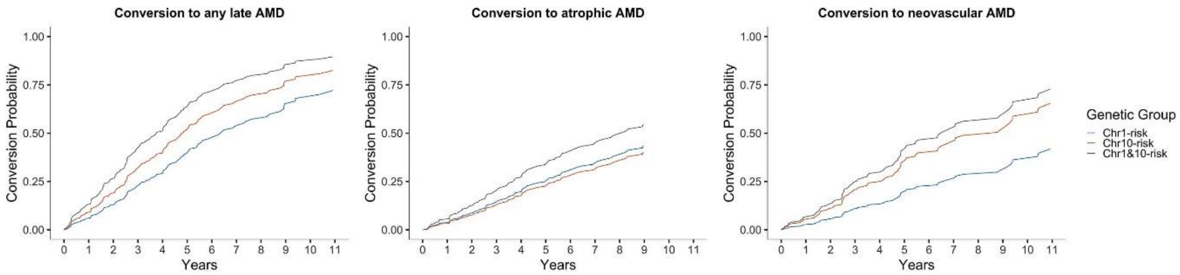

Survival curves for conversion to any late-stage AMD phenotype (*left*), atrophic late-stage (*middle*) and neovascular late-stage (*right*) in the *Chr1-ris*k (blue), *Chr10-risk* (orange) and *Chr1&10-risk* (green) groups, shown for the analyses including one eye per subject and adjusted for age at first visit and refined AMD grade at first visit. Data are shown for persons with 5 or fewer years between the last visit before the conversion visit and the actual visit of conversion (Tcon ≤ 5). Chr1-risk indicates homozygous for risk variants at chromosome 1 without risk at Chr10; Chr10-risk, homozygous for risk variants at chromosome 10 without risk at Chr1; and Chr1&10-risk, homozygous for risk variants at chromosomes 1 and 10.

.

© 2022 Schmitz-Valckenberg S et al. *JAMA Ophthalmol.*

eFigure 2. Survival Curves for Loss of Visual Acuity Based Upon T_{con} ≤ 5 Years

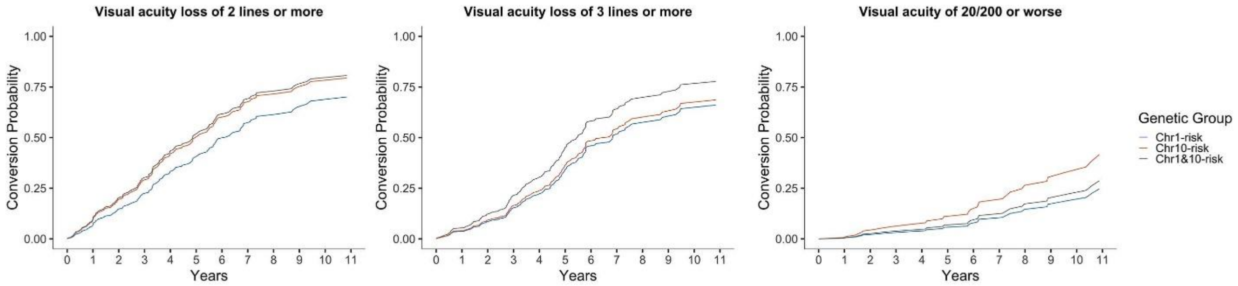

Survival curves for loss of visual acuity in the *Chr1-ris*k (blue), *Chr10-risk* (orange) and *Chr1&10-risk* (green) groups, shown for loss ≥ 2 lines (*left*), ≥ 3 lines (*middle*) and to 20/200 (*right*). The graphs are adjusted for age at first visit, refined AMD grade at first visit and visual acuity at first visit. Data are shown for persons with 5 or fewer years between the last visit before the conversion visit and the actual visit of conversion (Tcon ≤ 5). Chr1-risk indicates homozygous for risk variants at chromosome 1 without risk at Chr10; Chr10-risk, homozygous for risk variants at chromosome 10 without risk at Chr1; and Chr1&10-risk, homozygous for risk variants at chromosomes 1 and 10.

© 2022 Schmitz-Valckenberg S et al. *JAMA Ophthalmol.*

eTable 1. Genetic Diplotypes Employed to Define the Chr1-Risk, Chr10-Risk and Chr1&10-Risk Groups

**Genetic Profile**			**Chr1**	**Chr10**
**Group**	Chr1	Chr10	*CFH Y*402*H* rs1061170	*ARMS2/HTRA1* rs10490924
Chr1-risk	Risk	Non-risk	C/C	G/G
Chr10-risk	Non-risk	Risk	T/T	T/T
Chr1&10-risk	Risk	Risk	C/C	T/T

© 2022 Schmitz-Valckenberg S et al. *JAMA Ophthalmol.*

eTable 2. Comparison of Longitudinal Data for Eyes at Risk at the First Visit Between Genetic Groups on an Eye Level

Characteristic	*Chr1-risk*	*Chr10-risk*	*Chr1&10-risk*	***p*-value**
**Eyes (at risk at first visit)**				
No recorded conversion (censored)	292 (69.7%)	51 (55.4%)	51 (58.6%)	0.02[a] (X^2 = 1.6)
Conversion to atrophic AMD	79 (18.9%)	20 (21.8%)	19 (21.8%)	
Conversion to neovascular AMD	41 (9.8%)	18 (19.6%)	17 (19.6%)	
Exact conversion to neovascular late-stage not determinable	7 (1.7%)	3 (3.3%)	0	
**Age at Conversion**, median (IQR)				
Any late-stage	80.6 [75.1;85.2]	82.6 [75.4;84.5]	73.6 [70.3;77.6]	<0.001[b] (X^2 = 25.5)
Atrophic AMD*	81.2 [75.8;85.1]	83.0 [75.4;84.7]	73.7 [70.3.4;77.6]	<0.001[b] (X^2 = 16.8)
Neovascular AMD	80.2 [74.5;85.8]	80.3 [75.7;84.5]	73.6.3 [70.7;77.5]	0.01[b] (X^2 = 8.7)

[a]Pearson’s Chi-squared test. [b]Kruskall-Wallis rank sum test. IQR = Inter Quartile Range

© 2022 Schmitz-Valckenberg S et al. *JAMA Ophthalmol.*

eTable 3. Hazard Ratios for Visual Acuity Loss ≥ 2 Lines and ≥ 3 Lines

**Variable**	**Hazard Ratio (95%CI), *p -value***	
	VA loss ≥ 2 lines	VA loss ≥ 3 lines
**Genetic profile** *Chr1-risk* (reference)		
Chr10-risk	1.8 [1.0; 3.1], *p* = 0.05	1.5 [0.8; 2.7], *p* = 0.23
Chr1&10-risk	2.1 [1.1; 3.9], *p* = 0.03	1.9 [1.0; 3.8], *p* = 0.06
**Age at first visit**	1.1 [1.0; 1.1], *p* < 0.001	1.1 [1.0; 1.1], *p* = 0.007
**VA at first visit**	0.3 [0.08; 1.2], *p* = 0.09	0.1 [0.02; 0.8], *p* = 0.03
**Refined AMD grade** at first visit to medium-sized drusen (reference)	-	-
Large drusen and/or pigmentary changes	2.3 [1.2; 4.3], *p* = 0.009	2.3 [1.2; 4.5], *p* = 0.02
Large PED	3.2 [1.2; 8.7], *p* = 0.03	2.1 [0.6; 6.8], *p* = 0.2
iRORA	4.0 [1.7; 9.7], *p* = 0.002	4.8 [1.8; 12.6], *p* = 0.002

Abbreviations: Chr1-risk, homozygous for risk variants at chromosome 1 without risk at chromosome 10; Chr10-risk, homozygous for risk variants at chromosome 10 without risk at chromosome 1; Chr1&10, homozygous for risk variants at chromosomes 1 and 10; iRORA, incomplete retinal pigment epithelium and outer retinal atrophy; PED, pigment epithelium detachment; VA = visual acuity; 95% confidence intervals (CI) are shown in brackets, following hazards ratios and data refers to persons with 12 or fewer months between the last visit before the conversion visit and the actual visit of conversion.

© 2022 Schmitz-Valckenberg S et al. *JAMA Ophthalmol.*

eTable 4. Adjusted Median Survival Time For Visual Acuity Loss ≥ 2 Lines in Relation to the Refined AMD Stage at the First Visit, Separately Shown For Both Genetic Groups

	**Medium-sized drusen**	**Large drusen and/or pigmentary changes**	**Large PED**	**iRORA**
Chr1-risk	13.4 [11.2;*]	8.7 [6.7;*]	7.3 [4.8;*]	4.5 [2.8;*]
Chr10-risk	11.1 [6.7;*]	5.7 [3.8;*]	5.7 [2.7;*]	2.8 [1.7;9.4]
Chr1&10-risk	8.7 [5.7;*]	5.4 [3.3; 11.3]	4.1 [2.6;*]	2.6 [1.0;*]

Based on one eye per subject. Age and visual acuity at first visit were averaged among eyes sharing the same grade at the first visit. Abbreviations: asterisk (*),event rate did not reach 75%; iRORA, incomplete retinal pigment epithelium and outer retinal atrophy; PED, pigment epithelium detachment; Inter Quartile Ranges are given in brackets. Data is shown for persons with 12 or fewer months between the last visit before the conversion visit and the actual visit of conversion.

© 2022 Schmitz-Valckenberg S et al. *JAMA Ophthalmol.*

eReferences.

1. Team RC. R: A language and environment for statistical computing. R Foundation for Statistical Computing. Available online at https://www.R-project.org/. Published 2018. Accessed.

2. Therneau TM, Grambsch PM. *Modeling Survival Data: Extending the Cox Model.* New York: Springer; 2000.

3. Ripatti S, Palmgren J. Estimation of multivariate frailty models using penalized partial likelihood. *Biometrics.* 2000;56(4):1016-1022.

4. Therneau TM, Grambsch PM, Pankratz VS. Penalized Survival Models and Frailty. *Journal of Computational and Graphical Statistics.* 2003;12(1):156-175.

© 2022 Schmitz-Valckenberg S et al. *JAMA Ophthalmol.*
